# Carbapenem-resistant *Klebsiella pneumoniae* infections in Chinese children: *in vitro* activities of ceftazidime-avibactam and aztreonam-avibactam against carbapenemase-producing strains in a two-center study

**DOI:** 10.3389/fcimb.2025.1545999

**Published:** 2025-03-26

**Authors:** Xia Ran, Xue Chen, Cai Wang, Haiping Wang, Wei Xie, Chunmei Jing

**Affiliations:** ^1^ Department of Clinical Laboratory, Children’s Hospital of Chongqing Medical University, National Clinical Research Center for Child Health and Disorders, Ministry of Education Key Laboratory of Child Development and Disorders, Chongqing Key Laboratory of Child Rare Diseases in Infection and Immunity, Chongqing, China; ^2^ Department of Clinical Laboratory, Kunming Children’s Hospital, Kunming, China

**Keywords:** carbapenem-resistant *Klebsiella pneumoniae*, ceftazidime-avibactam, aztreonam-avibactam, KPC-2, NDM-1 and NDM-5, epidemiology characteristics, pediatric patient

## Abstract

**Objective:**

This study assessed epidemiology characteristics, carbapenem-resistance genes, and drug resistance to ceftazidime-avibactam (CZA) and aztreonam-avibactam (AZA) in children with carbapenem-resistant *Klebsiella Pneumoniae* (CRKP) infections.

**Methods:**

A total of 363 non-repetitive CRKP strains were collected from children who underwent two tertiary children’s hospital between 1 January 2021 and 30 June 2024 in Chongqing and Kunming in Southwest China. Carbapenem resistance genes and antimicrobial susceptibility were analyzed. Basic clinical characteristics of the patients were obtained from medical records.

**Results:**

bla_NDM-5_, bla_NDM-1,_ and bla_KPC-2_ were the predominant carbapenemase genes; their detection rates were 35.8%, 30.3%, and 25.3%, respectively. Patients in the KPC-2-producing *Klebsiella pneumoniae* (KPC-KP) (median age, 90 days) were older than those producing NDM-1 and NDM-5 *Klebsiella pneumoniae* (NDM-KP) (median age, 37 days) (*P < 0.05*). The detection rate of NDM-KP in the neonatal unit was higher compared with KPC-KP (62.5% vs. 9.8%, *P < 0.05*), while the detection rate of NDM-KP in the intensive care unit (ICU) was decreased compared with KPC-KP (9.6% vs. 40.2%, *P < 0.05*). NDM-KP had lower resistance rates to aminoglycosides and fluoroquinolones than KPC-KP; the resistance rate of aminoglycosides and fluoroquinolones among NDM-KP and KPC-KP in Chongqing was increased compared with Kunming. The sensitivity rates of KPC-KP to CZA and NDM-KP to AZA were 100%, and the MIC50 of the CRKP to CZA and AZA were 2 μg/mL and 0.125μg/mL, respectively.

**Conclusions:**

The epidemiological characteristics of Chinese children with CRKP infections, including the resistance genes and the antibiotic resistance of CRKP, exhibited significant variation between the two regions.KPC-KP strains had higher antimicrobial resistance in patients and thus should be given more attention in clinics and infection control.

## 1 Introduction

*Klebsiella pneumoniae* is the second leading pathogen responsible for clinical infectious diseases in China (Hu et al., 2024). It can cause infections in the respiratory system, urinary system, and various tissues, with a high morbidity and mortality rate (Hussein et al., 2013; Kontopoulou et al., 2019; Martin et al., 2018).Notably, the mortality rate among patients with *K.pneumoniae*-caused pneumonia is approximately 50% (Martin et al., 2018). The resistance rate of *Klebsiella pneumoniae* to meropenem has steadily increased from 2.9% in 2005 to 30.0% in 2023 (http://www.chinets.com/Data/GermYear) (Hu et al., 2024).Carbapenem-resistant *Klebsiella pneumoniae* (CRKP) has become widespread globally, leading to life-threatening infections. In terms of disability-adjusted life years (DALYs) per 100,000 population, the median for CRKP infections in the European Union is 11.5 (Karampatakis et al., 2023). An Australian study reported a pooled mortality rate of 37.2% due to CRKP infections (Agyeman et al., 2020). In Greece, the rate of CRKP isolates reached 66.3% in 2020, the highest among all countries (Cassini et al., 2019; European Centre for Disease Prevention and Control, 2019). CRKP is strongly associated with high mortality rates, particularly among critically ill and immunocompromised patients, especially young children, posing a significant threat to public health.CRKP is strongly associated with high rates of mortality, especially in critically ill and immunocompromised young patients, and poses immense threats to human health (Xu et al., 2017).In 2017, the World Health Organization (WHO) published a list of critical priority pathogens, including CRKP, for which there is an urgent need to develop new antibiotics (Nordmann and Poirel, 2019). Despite annual epidemiological surveillance reports on CRKP (Veeraraghavan and Walia, 2019; Yin et al., 2020), most previous studies focus on adult populations, while there is a lack of relevant literature focusing on children. Several multi-center studies conducted in different countries found differences in bacterial clones between pediatric and adult patients within the same centers (Castagnola et al., 2019). In China, the prevalence rate of CRKP is approximately 13.4% to 23% among children (Fu et al., 2021).

The production of carbapenemases, including Ambler A-class β-lactamase bla_KPC_, encoding metallo-β-lactamase bla_NDM_(B-class), and D-class β-lactamase bla_OXA-48_ (Lutgring, 2019), which are key mechanisms for carbapenem resistance in *Klebsiella pneumoniae.*In addition, studies have found that CRKP exhibits different molecular characteristics in children of different ages (Yin et al., 2020). For example, class A carbapenemase bla_KPC-2_ is mainly found in non-neonatal and adult patients, while class B carbapenemase bla_NDM-1_ is more prevalent in neonates (Yin et al., 2020). However, data on the clinical outcomes in children infected with CRKP are limited.

CRKP is usually resistant to the most commonly used antibiotics, and there are limited treatment options available. Since 2014, the development of new antibiotics brought new opportunities for treatments of CRKP. The novel approved antibiotics against CRKP infections include imipenem/cilastatin-relebactam, CZA, meropenem-vaborbactam, plazomicin, and eravacycline (Papp-Wallace, 2019). CZA includes a combination of a beta-lactam antibiotic and a beta-lactamase inhibitor that effectively inhibits the activity of class A, C, and some D class carbapenemases.Compared with other antibiotics, CZA can significantly improve the clinical survival rate (Theuretzbacher et al., 2021). In 2022, CZA was approved by the National Medical Products Administration for treating complicated intra-abdominal infections (cIAIs) in children older than 3 months. However, none of these β-lactamase-inhibitor combinations showed activity against carbapenemase, especially for metallo-β-lactamase (MBL) (Biagi et al., 2019). Still, the recent novel antibiotic aztreonam-avibactam (AZA) represented remarkable progress in treating MBL- or other β-lactamases-producing CRKP (Zou et al., 2020).

There have been very few studies on the activity of CRKP against CAZ and AZA in children. To the best of our knowledge, this is the first multicenter study that analyzed epidemiology characteristics of CRKP, genes related to CRKP, and drug resistance to ceftazidime-avibactam (CZA) and aztreonam-avibactam (AZA) in children. The present study also compared differences between genetic types and geographic regions. These data provide strong evidence for the clinical treatment, prevention, and control of CRKP in children.

## 2 Materials and methods

### 2.1 Bacterial strains

A total of 363 nonduplicate clinical CRKP isolates collected from patients who underwent two tertiary children’s hospital between 1 January 2021 and 30 June 2024 in Chongqing and Kunming in Southwest China were analyzed. Isolates identification and antimicrobial susceptibility testing (AST) were performed using the automated BD Phoenix™ M50 Microbiology System. P*. aeruginosa* ATCC27853 and *E. coli* ATCC 25922 were used as quality controls.

CRKP was defined as a resistant strain to any carbapenem antimicrobials (i.e., minimum inhibitory concentrations (MICs) of ≥ 2 μg/mL against ertapenem or ≥ 4 μg/mL against meropenem or imipenem. The results were interpreted by the breakpoint criteria recommended by the Clinical and Laboratory Standards Institute (CLSI) M100-S34 guidelines from 2024 (Clinical and Laboratory Standards Institute (CLSI), 2024).

### 2.2 Screening of carbapenemase genes

PCR was performed to screen the carbapenemase encoding genes, including *bla*_KPC_, *bla*_NDM_, *bla*_IMP_, *bla*_VIM,_ and *bla*_OXA-48-like_. Positive PCR products were sequenced by Sanger sequencing (Sangon Biotech), and the sequences were blasted in GenBank (https://blast.ncbi.nlm.nih.gov/Blast.cgi). The primers used for detecting these carbapenemase genes have been reported previously (Zhou et al., 2013; Wang et al., 2018).

### 2.3 *In vitro* antimicrobial susceptibility testing

The MICs of CRKP strains were determined using the standard broth microdilution method and were interpreted according to CLSI criteria (Clinical and Laboratory Standards Institute (CLSI), 2024). All CRKP strains were tested for MICs of CZA; only the NDM-KP strain detected MICs of AZA. For CZA and AZA testing, AVI was tested at a fixed concentration of 4 mg/L, while ceftazidime and aztreonam were added at different concentrations, respectively. The testing MICs range of CZA was 0.016/4–256/4 μg/mL. The testing MICs range of AZA was 0.032/4–64/4 μg/mL. MICs breakpoints for CZA were interpreted as CLSI guidelines (2024) (Clinical and Laboratory Standards Institute (CLSI), 2024). The MICs breakpoints for AZA were interpreted using the 2024 CLSI (Clinical and Laboratory Standards Institute (CLSI), 2024) breakpoints (interpretation via 2024 EUCAST criteria (EUCAST, 2024) is provided in Supplementary Table S2). P*. aeruginosa* ATCC27853 and *E. coli* ATCC 25922 were used as quality control strains. MICs were determined in triplicate on two separate days.

### 2.4 Definitions

Neonatal patients were defined as those no older than 28 days, while pediatric patients were defined as those between 29 days and 14 years old (Nichols et al., 2015).

### 2.5 Statistical analysis

Raw data were processed using Whonet 5.6 software and then calculated using GraphPad Prism 5. The age difference was further determined using the Mann-Whitney U test, and categorical data were evaluated using the *Chi-square* test or *Fisher’s* exact test. Statistical significance was confirmed if the two-tailed P-value was < 0.05.

## 3 Results

### 3.1 Carbapenem resistance gene of CRKP strains

**Figure 1:**
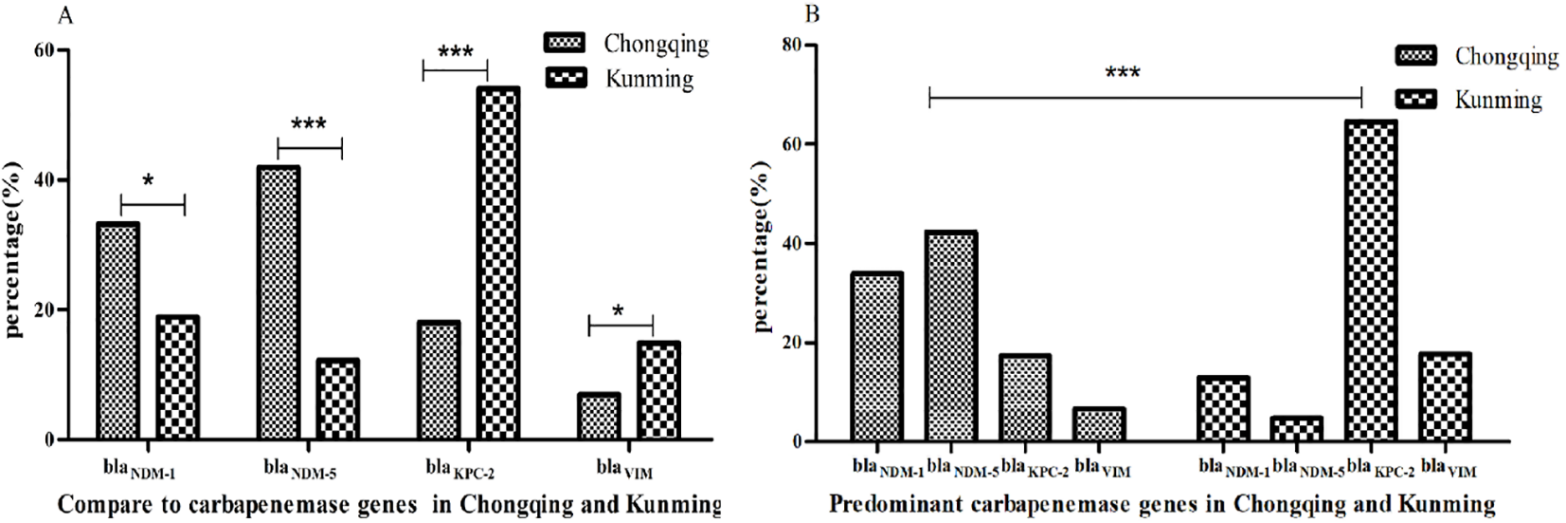
Resistance genes of CRKP isolates. **(A)** Compare to carbapenemase genes in Chongqing and Kunming. **(B)** Predominant carbapenemase genes in Chongqing and Kunming.**indicate statistical significance with P < 0.01, ***indicate statistical significance with P < 0.001.

Of the 363 CRKP isolates, 363 (100%) strains were successfully identified with the carbapenemase genes; bla_NDM-5_ (35.8%, 130/363) was a predominant gene, followed by bla_NDM-1_ (30.3%, 110/363), bla_KPC-2_ (25.3%, 92/363), and bla_VIM_ (8.5%, 31/363). The detection rates of bla_KPC-2_ and bla_NDM_ (bla_NDM-1_ and bla_NDM-5_) were 18.0%, 54.1%, and 75.1%, 31.1% in Chongqing and Kunming, respectively. Compared to Kunming, the detection rate of bla_NDM-5_ and bla_NDM-1_ increased while bla_KPC-2_ and bla_VIM_ decreased in Chongqing (all *P < 0.05*) (**Figure 1A**). In addition, bla_NDM-5_ was the most prevalent in Chongqing, while bla_KPC-2_ was the most prevalent in Kunming. Also, the detection rates of four carbapenem resistance genes were significantly different in Chongqing and Kunming (all *P < 0.05*) (**Figure 1B**).

### 3.2 Clinical characteristics and epidemiology of CRKP strains

A total of 363 non-repetitive CRKP strains were collected from pediatric inpatients. The clinical characteristics of these isolates are summarized in **Table 1**. The female-to-male ratio was 0.7, and the proportion of males was 63.0% and 61.7% in KPC-KP and NDM-KP, respectively. Patients who were colonized or infected with CRKP had a median age of 48 days (interquartile range, 1–6120 days). Of the 363 CRKP isolates, 57.6% (n = 209) were collected from sputum, 14.6% (n = 53) from blood samples, and 5.5%% (n = 20) from urine samples. Patients in the KPC-KP (median age, 90 days) were older than the NDM-KP (median age, 37 days) (*P < 0.05*) (**Table 1**). Most CRKP strains were isolated from the neonatal unit (41.9%) and ICU (22.0%); most NDM-KP were collected from the neonatal unit (62.5%, 150/240), and isolates carrying KPC-2 were mainly detected in the ICU (40.2%, 37/92). The detection rate of NDM-KP in the neonatal unit was increased compared with KPC-KP (62.5% vs. 9.8%, *P < 0.05*). However, the detection rate of NDM-KP in the ICU was decreased compared with KPC-KP (9.6% vs. 40.2%, *P<0.05*). In addition, the detection rates of bla_NDM-1_ and bla _NDM-5_ were 57.9% (92/159) and 36.5% (58/159) in the neonatal unit, respectively. NDM-KP and KPC-KP detection rates were the highest in 2022 and 2024, respectively (**Table 1**).

**Table 1:** Clinical characteristics of the CRKP strains.

	Total (n = 363)	KPC-KP (n = 92)	NDM-KP (n = 240)	P-value
Male sex	216 (59.5%)	58 (63.0%)	148 (61.7%)	0.817
Age in days (median)	48	90	37	<0.0001
Specimen				
Sputum	209 (57.6%)	52 (56.5%)	146 (60.8%)	0.474
Blood	53 (14.6%)	13 (14.1%)	38 (15.8%)	0.700
Urine	20 (5.5%)	9 (9.8%)	13 (5.4%)	0.152
Others	81 (22.3)	18 (19.6%)	43 (17.9%)	0.729
Isolation wards				
Neonatal unit	152 (41.9%)	9 (9.8%)	150 (62.5%)	<0.0001
Intensive care unit	80 (22.0%)	37 (40.2%)	23 (9.6%)	<0.0001
Others	131 (36.1%)	46 (50.0%)	67 (27.9%)	0.0001
Years				
2021	83 (25.0%)	9 (9.8%)	74 (30.8%)	<0.0001
2022	102 (30.7%)	8 (8.7%)	94 (39.2%)	0.0001
2023	91 (27.4%)	29 (31.5%)	62 (25.8%)	0.2983
2024	56 (16.9%)	46 (50.0)	10 (4.2%)	<0.0001

### 3.3 Antimicrobial susceptibility testing

As shown in **Table 2**, all CRKP isolates were defined as MDR for resistance to more than three antibiotic classes. Also, all strains showed high resistance to cephalosporin antibiotics (100%). The resistance rates to aztreonam (88.3%) were higher than 70.0%. The resistance rates to amikacin, gentamicin, and trimethoprim-sulfamethoxazole were 29.4%, 35.4%, and 34.1%, respectively. The percentage of resistance to ciprofloxacin and levofloxacin was 39.8% and 34.0%, respectively. Of note, the KPC-KP group showed a different antibiotic resistance spectrum to the non-β-lactams than the NDM-KP group. The MIC50 of gentamicin, ciprofloxacin, and levofloxacin in the KPC-KP group (16, 4, 8, respectively) was higher than in the NDM-KP group (2, 0.25, 0.5, respectively). Compared with the KPC-KP group, the NDM-KP group had lower resistance rates to amikacin (2.1% vs. 89.8%), gentamicin (2.1% vs. 96.2%), ciprofloxacin (14.2% vs. 96.3%), levofloxacin (5.9% vs. 96.3%) and aztreonam (85.4% vs. 100%) (all *P < 0.05*, **Table 2**).

**Table 2:** Antimicrobial activities tests of CRKP strains.

Antibiotics	Total (n = 363)			KPC-KP (n = 92)			NDM-KP (n = 240)			P-value (KPC-KP R%/NDM-KP R%)
	MIC50	MIC90	R%	MIC50	MIC90	R%	MIC50	MIC90	R%	
CZO	32	32	100	32	32	100	32	32	100	–
CXM	32	64	100	64	64	100	32	64	100	–
CTX	64	64	100	64	64	100	64	64	100	–
CRO	64	64	100	64	64	100	64	64	100	–
CAZ	32	64	100	64	64	100	32	64	100	–
FEP	32	32	100	32	32	100	32	32	100	–
AMC	32	64	100	32	64	100	32	64	100	–
SAM	32	32	100	32	32	100	32	32	100	–
CSL	64	64	100	64	64	100	64	64	100	–
TZP	128	128	99.4	128	128	100	128	128	99.2	0.3798
ETP	4	8	97	8	8	98	4	8	96.1	0.4727
MEM	16	16	100	16	16	100	16	16	100	–
IPM	16	16	100	16	16	100	16	16	100	–
AMK	8	64	29.4	64	64	89.8	8	8	2.1	<0.0001
GEN	2	16	35.4	16	16	96.2	2	16	2.1	<0.0001
CIP	0.5	8	39.8	4	8	96.3	0.25	1	14.2	<0.0001
LVX	0.5	16	34.0	8	16	96.3	0.5	1	5.9	<0.0001
SXT	1	8	34.1	1	8	41.7	1	16	30.7	0.0709
ATM	32	64	88.3	64	64	100	32	64	85.4	0.0001

CZO, cefazolin; CXM, cefuroxime; CTX, cefotaxime; CRO, ceftriaxone; CAZ, ceftazidime; FEP, cefepime; AMC, amoxicillin-clavulanate; SAM, ampicillin-sulbactam; CSL, ceftazidime-sulbactam; TZP, piperacillin-tazobactam; ETP, ertapenem; MEM, meropenem; IPM, imipenem; AMK, amikacin; GEN, gentamicin; CIP, ciproﬂoxacin; LVZ, levofloxacin; SXT, sulfamethoxazole-trimethoprim; ATM, aztreonam; CZA, ceftazidime- avibactam; AZA, aztreonam-avibactam.

As shown in **Table 3**, the KPC-KP strains showed differences in antibiotic resistance rates between Chongqing and Kunming; the resistance rate of ertapenem in Chongqing was lower than that in Kunming, while the resistance rates to amikacin, gentamicin, ciprofloxacin, and levofloxacin in Chongqing were higher than those in Kunming. The MIC50 of sulfamethoxazole-trimethoprim in Chongqing (8) was higher than in Kunming (1), Chongqing also showed greater resistance to sulfamethoxazole-trimethoprim than Kunming (*P < 0.05*, **Table 3**).

**Table 3:** Antimicrobial activities tests of KPC-KP strains.

Antibiotics	Chongqing (n = 52)			Kunming (n = 40)			P-value (Chongqing R%/Kunming R%)
	MIC50	MIC90	R%	MIC50	MIC90	R%	
CZO	32	32	100	32	32	100	–
CXM	32	32	100	64	64	100	–
CTX	64	64	100	64	64	100	–
CRO	64	64	100	64	64	100	–
CAZ	64	64	100	32	64	100	–
FEP	32	32	100	32	32	100	–
AMC	64	64	100	32	32	100	–
SAM	32	32	100	32	32	100	–
CSL	64	64	100	64	64	100	–
TZP	128	128	100	128	128	100	–
ETP	4	4	95.3	8	8	100	0.2098
MEM	16	16	100	16	16	100	–
IPM	16	16	100	16	16	100	–
AMK	64	64	96.2	64	64	83.9	0.0598
GEN	16	16	96.2	16	16	83.9	0.0598
CIP	8	8	98.1	4	4	94.6	0.4101
LVX	16	16	98.1	8	8	94.6	0.4101
SXT	8	8	71.2	1	16	14.3	<0.0001
ATM	64	64	100	64	64	100	–

CZO, cefazolin; CXM, cefuroxime; CTX, cefotaxime; CRO, ceftriaxone; CAZ, ceftazidime; FEP, cefepime; AMC, amoxicillin-clavulanate; SAM, ampicillin-sulbactam; CSL, ceftazidime-sulbactam; TZP, piperacillin-tazobactam; ETP, ertapenem; MEM, meropenem; IPM, imipenem; AMK, amikacin; GEN, gentamicin; CIP, ciproﬂoxacin; LVZ, levoﬂoxacin; SXT, sulfamethoxazole-trimethoprim; ATM, aztreonam; CZA, ceftazidime- avibactam; AZA, aztreonam-avibactam.

As shown in **Table 4**, the NDM-KP strains showed differences in antibiotic resistance rates between Chongqing and Kunming.Compared with Kunming, the lower resistance rates to piperacillin-tazobactam, ertapenem and aztreonam were found in Chongqing, while the resistance rates to amikacin, gentamicin, ciprofloxacin, and levofloxacin in Kunming were lower than those in Chongqing. The MIC50 of sulfamethoxazole-trimethoprim in Chongqing (1) was lower than in Kunming (16), while the resistance rates to sulfamethoxazole-trimethoprim in Chongqing was lower than those in Kunming(P<0.05, **Table 4**).

**Table 4:** Antimicrobial activities tests of NDM-KP strains.

Antibiotics	Chongqing (n = 217)			Kunming (n = 23)			P-value (Chongqing R%/Kunming R%)
	MIC50	MIC90	R%	MIC50	MIC90	R%	
CZO	32	32	100	64	64	100	–
CXM	32	32	100	64	64	100	–
CTX	64	64	100	64	64	100	–
CRO	64	64	100	64	64	100	–
CAZ	32	64	100	64	64	100	–
FEP	32	32	100	32	32	100	–
AMC	32	64	100	32	32	100	–
SAM	32	32	100	32	32	100	–
CSL	64	64	100	64	64	100	–
TZP	128	128	99.1	128	128	100	0.6438
ETP	4	8	95.2	8	8	100	0.2930
MEM	16	16	100	4	4	100	–
IPM	16	16	100	16	16	100	–
AMK	8	8	2.3	2	2	0	0.4619
GEN	2	16	21.2	1	1	0	0.0141
CIP	0.25	1	14.7	0.125	0.5	9.1	0.4288
LVX	0.5	1	6.0	1	1	4.5	0.7492
SXT	1	8	25.9	16	16	77.3	<0.0001
ATM	32	64	85.3	64	64	100	0.0479

CZO, cefazolin; CXM, cefuroxime; CTX, cefotaxime; CRO, ceftriaxone; CAZ, ceftazidime; FEP, cefepime; AMC, amoxicillin-clavulanate; SAM, ampicillin-sulbactam; CSL, ceftazidime-sulbactam; TZP, piperacillin-tazobactam; ETP, ertapenem; MEM, meropenem; IPM, imipenem; AMK, amikacin; GEN, gentamicin; CIP, ciproﬂoxacin; LVZ, levoﬂoxacin; SXT, sulfamethoxazole-trimethoprim; ATM, aztreonam; CZA, ceftazidime- avibactam; AZA, aztreonam-avibactam.

**Figure 2:**
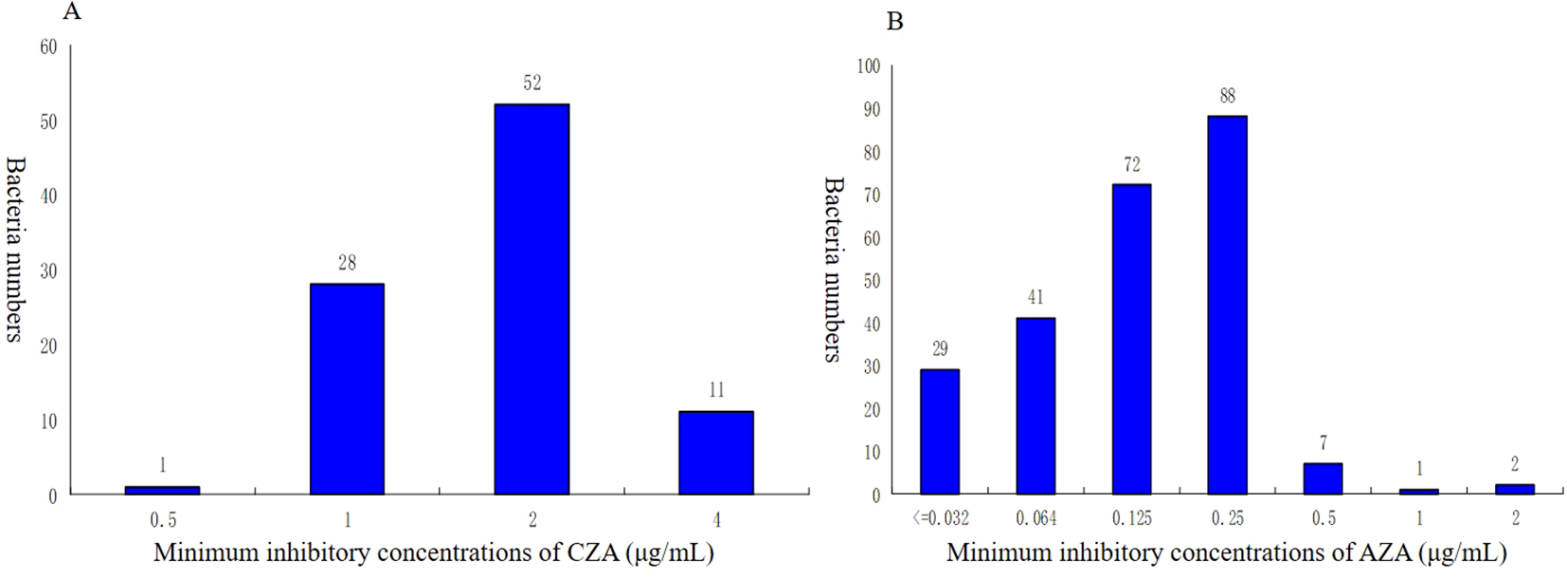
Distribution of minimum inhibitory concentrations (MIC) for CZA and AZA of CRKP. **(A)** Minimum inhibitory concentrations of CZA (µg/ml). **(B)**Minimum inhibitory concentrations of AZA (µg/ml). CZA, ceftazidime-avibactam; AZA, aztreonam-avibactam.

The resistance rates to CZA (74.7%) were higher than 70.0%. Moreover, a much lower level of resistance to AZA (0%) was observed in this study. Additionally, The MIC50 of CZA in the KPC-KP group (2) was lower than that in the NDM-KP group (128). In addition, NDM-KP strains had greater resistance to CZA than the KPC-KP group (*P<0.05*). MICs for CZA isolates ranged from 0.5 to 4 µg/mL; MIC50 and MIC90 of KPC-KP strains were 2 and 4 µg/mL, and MICs for AZA isolates ranged from ≤ 0.032 to 2 µg/mL. MIC50 and MIC90 of NDM-KP strains were 0.125 and 0.25 µg/mL, respectively (**Tables 5**, **6**, **Figure 2**).

**Table 5:** Antimicrobial activities tests of CZA.

	CRKP strains			KPC-KP strains		NDM-KP strains	
	Total (n = 363)	KPC-2 (n = 92)	NDM* (n = 240)	Chongqing (n = 52)	Kunming (n = 40)	Chongqing (n = 217)	Kunming (n = 23)
MIC50	128	2	128	2	2	128	128
MIC90	128	4	128	2	4	128	128
R%	74.7	0	100	0	0	100	100

**Table 6:** Antimicrobial activities tests of AZA.

	CRKP strains			NDM-KP strains	
	Total (n = 363)	KPC-2 (n = 92)	NDM* (n = 240)	Chongqing (n = 217)	Kunming (n = 23)
MIC50	0.125	–	0.125	0.125	0.125
MIC90	0.25	–	0.25	0.25	0.25
R%	0	–	0	0	0

## Discussion

The epidemiological study of CRKP infection is vital for developing clinical treatment strategies and evaluating the effectiveness of different treatment approaches. Regional differences in the distribution of bacteria exist due to variations in climate, economy, and medical conditions. Regional variations in CRKP prevalence have been reported (Wyres and Holt, 2022). Children, as a special population, have underdeveloped organs, relatively lower immune function, and are therefore more susceptible to bacterial infections. Furthermore, the composition and resistance profiles of pathogenic bacteria differ between children and adults. Understanding the resistance patterns and regional variations of antibiotics is thus of great importance.Currently, data on the clinical and epidemiology of CRKP infection with different resistant gene types are limited in children. This is the first multicenter study on the epidemiological characteristics of CRKP in children, its associated genes, and resistance to ceftazidime-avibactam (CZA) and aztreonam-avibactam (AZA), comparing differences across genetic types and geographic regions. Some studies (Castagnola et al., 2019) detected differences in bacterial clones among pediatric and adult patients within the same centers. The prevalent carbapenem-resistant gene in Chinese adults was bla_KPC_,while the prevalent carbapenem-resistant genes widely vary in pediatric patients (Wang et al., 2018; Li et al., 2022). Domestic and international studies have shown that bla_NDM_ is the most common carbapenemase gene in the pediatric population (Zhou et al., 2022; Ilham et al., 2023). Early studies (Wang et al., 2018) reported that bla_NDM-5_ is rarely found in *Klebsiella pneumoniae* in China. In the present study, the most frequently detected carbapenemase was bla_NDM-5_ (35.8%), and the primary carbapenem resistance gene was bla_NDM-5_ and bla_KPC-2_ in the Chongqing and Kunming, respectively, which may be due to regional variations of CRKP (Wyres and Holt, 2022). We inferred that the frequent switch of predominant carbapenemase genotype might result from the introduction of those strains from different sources (like regions, age, or specimen) with different prevalent carbapenemase genes or transformation of some mobile elements carrying carbapenemase genes that underwent transfers between species (Marques et al., 2019; Zhang et al., 2016; Zhang et al., 2018). Although the underlying mechanism remains unclear, this further emphasizes the importance of active resistance monitoring for CRKP in pediatric patients.

Differences were also observed in the distribution of NDM-KP and KPC-KP among different departments and patients of different ages. A total of 363 CRKPs were concentrated in the neonatal unit and ICU (63.9%). The average age of CRKP detection was 48 days, which is consistent with previous literature reports (Flannery et al., 2022; Yin et al., 2021). Our results showed that patients in the KPC-KP were older than those NDM-KP (90 days vs. 37 days, *P < 0.05*). In this study, the detection rate of bla_NDM-1_ was 57.9% in the neonatal unit, which is consistent with the molecular epidemiological studies of CRKP(bla_NDM-1_ is the main resistance mechanism of CRKP strains of neonatal) (Qin et al., 2014; Yin et al., 2020; Yin et al., 2021). CRKP-producing NDM was mainly distributed in younger neonates while CRKP-producing KPC-2 was mainly found in older non-neonates. Noteworthy, we observed a trend of the emergence of carbapenem resistance gene with CRKP from carrying NDM to KPC-2 in Chinese children. Bla_KPC-2_ is the most common A-class β-lactamase, with stronger transmission ability and higher toxicity than other carbapenemase genes (Deshpande et al., 2006; Nordmann et al., 2009). It is more commonly found in older children and adult patients. In fact, there have been many hospital outbreak reports at home and abroad (Gaspar et al., 2022; Huang et al., 2022; Maltezou et al., 2009).

Our results showed that CRKP was a multi-drug-resistant bacterium, with resistance rates of > 95% to first, second, and third-generation cephalosporins, enzyme inhibitors, and carbapenems, indicating a dire situation of drug resistance, which is consistent with previous studies (Cienfuegos-Gallet et al., 2022; Lei et al., 2022). However, the sensitivity to aminoglycosides and fluoroquinolones was higher, which may be due to the limited use of these antibiotics in pediatric patients due to renal, ear, and cartilage toxicity. When facing the problem of multi-drug resistance can still be treated with those antibiotics. Currently, it is believed that the treatment of CRKP infections is better with combined drug therapy than when using single drugs. Although there is a lack of research on the treatment of children, the treatment for adult patients can be used as a helpful reference; however, adjustments should be made in terms of drug dosage and variety. Our findings also suggested that alternating the use of antibiotics and strengthening the rational use of antibiotics can partially restore the sensitivity of antibiotics. We observed different resistance patterns in KPC-KP and NDM-KP, which is similar to the results of other pediatric studies (Liao et al., 2020). Compared to NDM-KP, KPC-KP showed more severe resistance to antibiotics such as amikacin, gentamicin, ciprofloxacin, levofloxacin, and sulfamethoxazole-trimethoprim, which should be taken seriously by clinicians and infection control professionals.

The antibiotic susceptibility results revealed significant differences in KPC-KP and NDM-KP across different regions. In Kunming, the resistance rates of CRKP to amikacin, gentamicin, ciprofloxacin, and levofloxacin were lower than those in Chongqing. This finding suggests that antimicrobial resistance exhibits regional variation, which is consistent with other studies reporting significant regional variations in bacterial characteristics, clinical outcomes, and antimicrobial resistance in global CRKP epidemics (Wang et al., 2022).Interestingly, for the KPC-KP, the resistance rate to sulfamethoxazole-trimethoprim was significantly higher in the Chongqing than in the Kunming, while for the NDM-KP, it was significantly lower. Sulfamethoxazole-trimethoprim has a high sensitivity, probably due to potential adverse reactions such as allergic reactions and liver and kidney function damage in children, which may limit its use. When facing the problem of multi-drug resistance and economic pressure, tuberculosis can still be treated with sulfamethoxazole-trimethoprim in combination with other drugs.

CRKP can be caused by different mechanisms, among which the production of carbapenemases is the most common. The Ambler class A (e.g., KPC), Ambler class B (e.g., NDM), and Ambler class D (e.g., OXA-48-like) carbapenemases are the three major classes of carbapenemases. Class A carbapenemases have serine-based hydrolytic activity (Bush, 2018; Lee et al., 2016).These enzymes are primarily KPC carbapenemases, whose activity can be inhibited by avibactam. The enzyme-producing strains are typically sensitive to CZA. Metallo-β-lactamase (MBL) enzymes (which encompass the Ambler class B enzymes) require the presence of metal for their activity (Bush, 2018; Lee et al., 2016).These enzymes are primarily NDM carbapenemases, and their activity cannot be inhibited by avibactam. Few of the enzyme-producing strains are sensitive to aztreonam. Therefore, distinguishing between KPC- and NDM-producing strains is crucial for the development of effective therapeutic strategies. In recent years, the availability of several novel β-lactam/β-lactamase inhibitor combinations has given hope for the clinical treatment of CRKP. In this study, drug susceptibility tests were performed for CZA and AZA. CZA is an intravenously administered combination of the third-generation cephalosporin Ceftazidime and the novel, non-β-lactam β lactamase inhibitor avibactam. It exhibits excellent *in vitro* activity against various significant gram-negative pathogens, including numerous enterobacteriaceae producing OXA-48, AmpC, and extended-spectrum β-lactamases. However, some reports have documented CZA resistance to KPC-KP in China (Zhou et al., 2024; Zou et al., 2019). The present study found the resistance rates of CRKP to CZA of 74.7%, which is higher than the 36.0% reported by Dan Li (Li et al., 2023). The difference in susceptibility rates of CRKP to CZA may be attributed to the different types of carbapenemase produced. This study found that all KPC-KP strains were sensitive to CZA due to the ability of avibactam to inhibit KPC enzyme activity. We tested CZA resistance in MBL-producing strains to verify its efficacy in treating pediatric CRKP infections. The results showed that all MBL-producing strains (NDM-KP) were resistant to CZA, as CZA is ineffective against MBL-producing strains due to the inability of avibactam to inhibit the activity of metallo-β-lactamase (MBL) enzymes. The MIC50 of CZA in KPC-KP strains (2) was higher than the value of 1 reported in adult patients by Chunhong Zou (Zou et al., 2020). Therefore, it is crucial to consider age and regional variations to establish baseline data for future treatments, and to strengthen monitoring of CZA drug resistance.As AZA may simultaneously target different types of carbapenemases, it could theoretically be used to treat CRKP infections that produce variable carbapenemases. This study showed that all the studied NDM-KP were 100% sensitive to AZA (MIC50 = 0.125 mg/L, MIC90 = 0.25 mg/L) *in vitro*, which is lower than the MIC50 = 1 mg/L and MIC90 = 4 mg/L reported by Dan Li (Li et al., 2023).Aztreonam is the only clinically used β-lactam antibiotic stable to MBL hydrolysis. As Enterobacteriaceae carrying MBLs may frequently harbor additional aztreonam-inactivating β-lactamases, the activity of aztreonam against these isolates is often compromised. However, the addition of avibactam to aztreonam makes this combination effective against MBL producers (Das et al., 2019; Li et al., 2015). Therefore, AZA has been proposed as a treatment for infections caused by MBL producers. A study reported that blaNDM is the most common carbapenemase gene in the pediatric population (Ilham et al., 2023; Zou et al., 2022). Consequently, the use of AZA for treating CRKP infections in the pediatric population is crucial.

This study has two limitations. Firstly, ceftazidime-avibactam, meropenem-vaborbactam, and imipenem-relebactam are three novel antimicrobial agents used for the treatment of KPC-producing *Klebsiella pneumoniae*. Cefiderocol is a novel siderophore cephalosporin targeting Gram-negative bacteria, including strains with NDM-producing *Klebsiella pneumoniae*. However, meropenem-vaborbactam, imipenem-relebactam, and cefiderocol are not available in China, and thus, we did not test them. Secondly, phylogenetic analysis was not performed on all carbapenem-resistant *Klebsiella pneumoniae* strains.

In conclusion, we analyzed the clinical characteristics, carbapenem resistance gene, and resistance to CZA and AZA of 363 CRKP in Chinese children, as well as the differences between different regions and carbapenem gene, identifying bla_NDM-5_, bla_NDM-1,_ and bla_KPC-2_ as the primary resistance genes. There were differences in carbapenem resistance gene and antibiotic resistance rates among different regions. KPC-KP and NDM-KP showed different clinical and molecular epidemiological characteristics, with KPC-KP showing more severe resistance, thus posing a more serious challenge to hospital infection control.AZA is preferentially used in regions with a high prevalence of NDM-KP, particularly in the pediatric population. Therefore, it is essential to tailor treatments based on resistance profiles and to strengthen the monitoring of drug resistance in CRKP infections in children, enabling clinicians to effectively treat CRKP infections and curb the global spread of drug-resistant bacteria.

## Data Availability

The datasets presented in this study can be found in online repositories. The names of the repository/repositories and accession number(s) can be found in the article/supplementary material.
